# Supplementary material for: Karyotype Description and Comparative Chromosomal Mapping of 5S rDNA in 42 Species
Source: Genes (Basel). 2024 May 20;15(5):647. doi: 10.3390/genes15050647 (PMC11121585; doi:10.3390/genes15050647)
Supplement: Supplementary file 1 [file genes-15-00647-s001.zip › Supplementary Table S1.pdf]

Supplementary Table S1. 5S rDNA as a FISH probe was an excellent marker to label plant chromosome in previous reaches.

Family	Species	Species Number	Reference
Brassicaceae	*Aethionema schistosum* Boiss. & Kotschy	1	Ali et al. 2005
	Alyssum spinosum L.	1	
	Arabidopsis (DC.) Heynh. f	4	
	Arabis L.	8	Ali et al. 2005, Robledo and Seijo 2008, Custódio et al. 2013
	Brassica L.	17	Ali et al. 2005, Campomayor et al. 2021, Hasterok et al. 2005, Kulak et al. 2002
	Cakile maritima Scop. subsp. *maritima*	1	Ali et al. 2005
	Camelina microcarpa Andrz. ex DC.	1	
	Capsella rubella Reut	1	
	Cardaminopsis carpatica Mesicek nom. prov	1	
	Carrichtera annua (L.) DC.	1	
	Chrysochamela velutina (DC.) Boiss.	1	
	Conringia orientalis (L.) Dumort.	1	
	Diplotaxis muralis (Linnaeus) de Candolle	2	Hasterok et al. 2005
	Eruca Mill.	2	Ali et al. 2005, Hasterok et al. 2005
	Erucastrum gallicum (Wild.) O.E. Schulz	1	Ali et al. 2005
	Hirschfeldia incana (L.) Lagr. Foss.	1	
	Iberis L.	2	
	Crucihimalaya wallichii (Hook. f. & Thoms.) Al-Shehbaz, O' Kane & Price	1	
	Moricandia arvensis (L.) DC.	1	
	Myagrum perfoliatum L.	1	
	Neslia paniculata (L.) Desv.	1	
	Olimarabidopsis Al-Shehbaz, O'Kane & R. A. Price	2	
	Psychine stylosa Desf.,	1	
	Rapistrum rugosum (L.) J.P. Bergeret	1	
	Raphanus L.	3	Campomayor et al. 2021, Hasterok et al. 2005
	Ricotia cretica Boiss. & Heldr.,	1	Ali et al. 2005
	Rorippa palustris (L.) Besser	1	
	Sinapidendron frutescens (Sol.) Lowe	1	
	Sinapis L.	3	Ali et al. 2005, Hasterok et al. 2005
	Sisymbrium officinale L.	1	Ali et al. 2005
	Thlaspi L.	2	
		66	

Asteraceae	*Chrysanthemum* L.	11	56	He et al. 2022d
	Coreopsis major Watt.	1		Garcia et al. 2010
	Crepis L.	41		Senderowicz et al. 2022
	Tagetes patula L.	1		
	Tripleurospermum maritimum (L.) W.D.J. Koch	1		Garcia et al. 2010
	Xerochrysum bracteatum (Ventenat) Tzvelev	1		
Fabaceae	*Amorpha fruticose* L. *	1	44	He et al. 2022
	Lotus japonicus Gifu	2		Pedrosa et al. 2002
	Macroptilium (Benth.) Urban	6		de Barros et al. 2023
	Onobrychis Mill.	29		Yucel et al. 2022
	Piptanthus concolor Harrow ex Craib*	1		Luo et al. 2017
	Styphnolobium japonicum (L.) Schott*	1		He et al. 2022
	Robinia L. *	3		
	Trifolium medium L.	1		Lukjanová et al. 2023
Orchidaceae	*Paphiopedilum* Pfitzer	37	37	Lan and Albert 2011
Cucurbitaceae	*Cucumis* L.	20	20	Zhang et al. 2016
Passifloraceae	*Passiflora* L.	20	20	de Melo and Guerra 2003
Convolvulaceae	*Cuscuta* L.	10	10	Ibiapino et al. 2022
Poaceae	*Deschampsia* P. Beauv.	3	10	Amosova et al. 2022
	Hordeum L.	7		Taketa et al. 2001, Waminal et al. 2018
Solanaceae	*Atropa belladonna* L.	1	8	Volkov et al. 2017
	Nicotiana L.	7		Kitamura et al. 2005
Byblidaceae	*Byblis* Salisb.	7	7	Fukushima et al. 2011
Pinaceae	*Pinus* L. *	5	5	Cai et al. 2006
Asparagaceae	*Asparagus* L.	5	5	Plath et al. 2022
Oleaceae	*Fraxinus pennsylvanica* Marsh. *	1	4	Luo and Liu 2019
	Ligustrum L. *	2		
	Syringa oblata Lindl. *	1		
Iridaceae	*Iris* L.	3	3	Park et al. 2022
Lamiaceae	*Salvia* L.	3	3	Muravenko et al. 2022
Berberidaceae	*Berberis* L. *	2	2	Liu and Luo 2019
Malvaceae	*Adansonia digitata* L. *	1	2	Islam-Faridi et al. 2020
	Hibiscus mutabilis L. *	1		Luo and He 2021
Plantaginaceae	*Plantago maxima* Juss. ex Jacq.	1	1	Kovács et al. 2023
Polemoniaceae	*Polemonium caeruleum* L.	1	1	Samatadze et al. 2023
Rutaceae	*Zanthoxylum armatum* DC. *	1	1	Luo et al. 2018, He et al. 2023

Note: asterisk (*) in supplementary Table 1 indicates 5S rDNA as a FISH probe was an excellent marker to label 20+ woody plants species.
