# Supplementary figures and images for: Karyotype Description and Comparative Chromosomal Mapping of 5S rDNA in 42 Species
Source: Genes (Basel). 2024 May 20;15(5):647. doi: 10.3390/genes15050647 (PMC11121585; doi:10.3390/genes15050647)

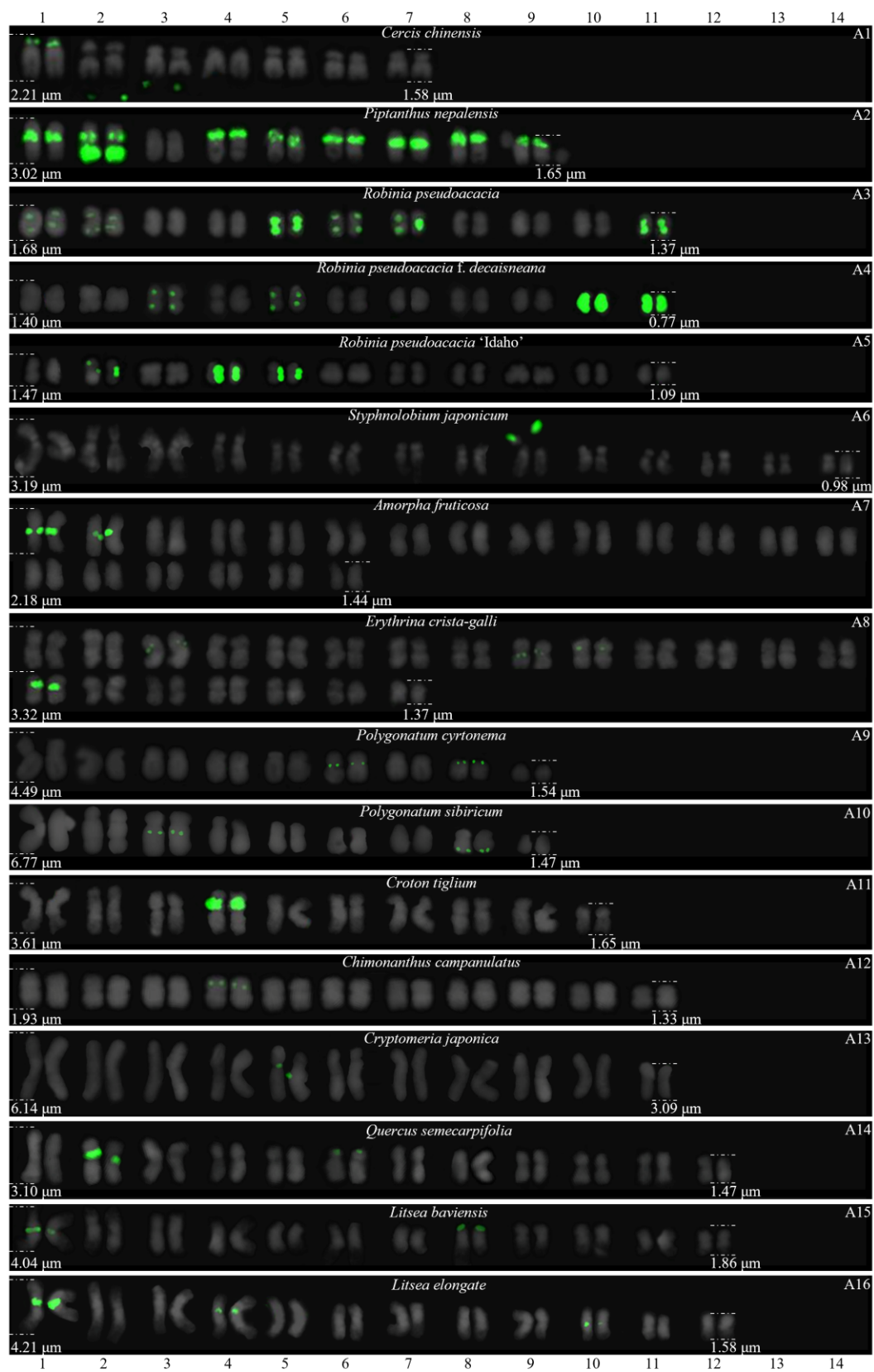

Supplement: Supplementary file 1 [file genes-15-00647-s001.zip › Supplementary Figure S1.pdf]

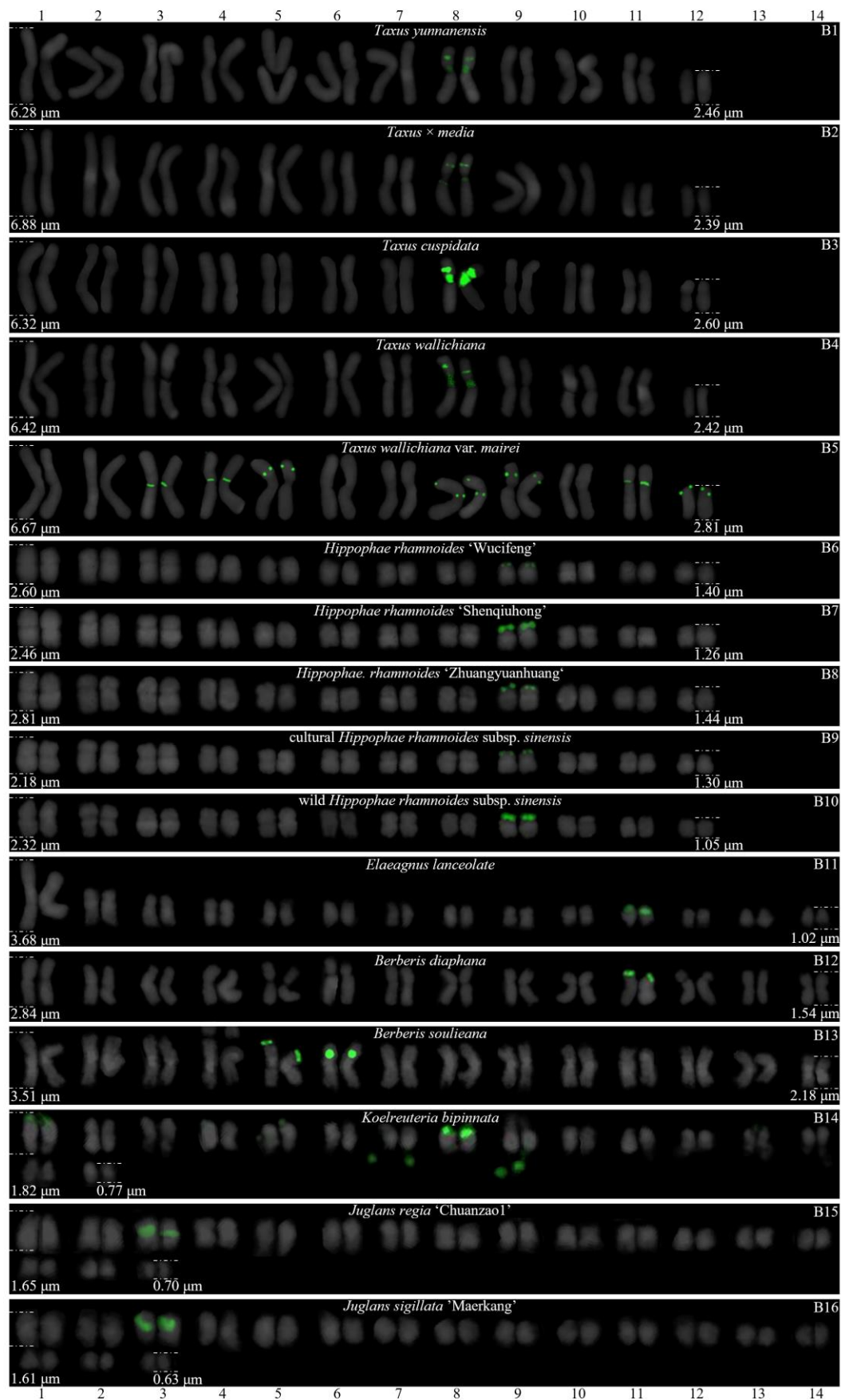

Supplement: Supplementary file 1 [file genes-15-00647-s001.zip › Supplementary Figure S2.pdf]

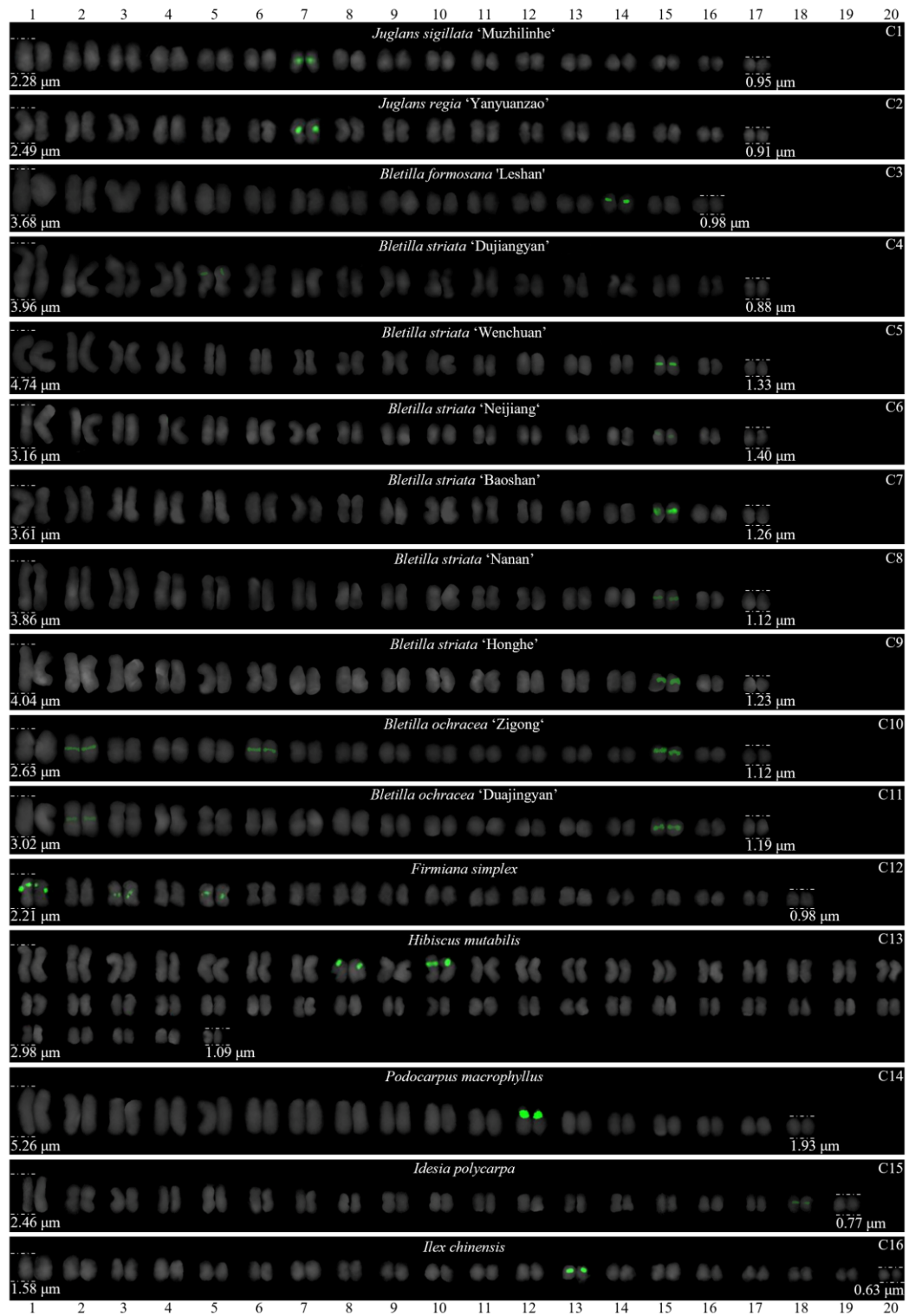

Supplement: Supplementary file 1 [file genes-15-00647-s001.zip › Supplementary Figure S3.pdf]

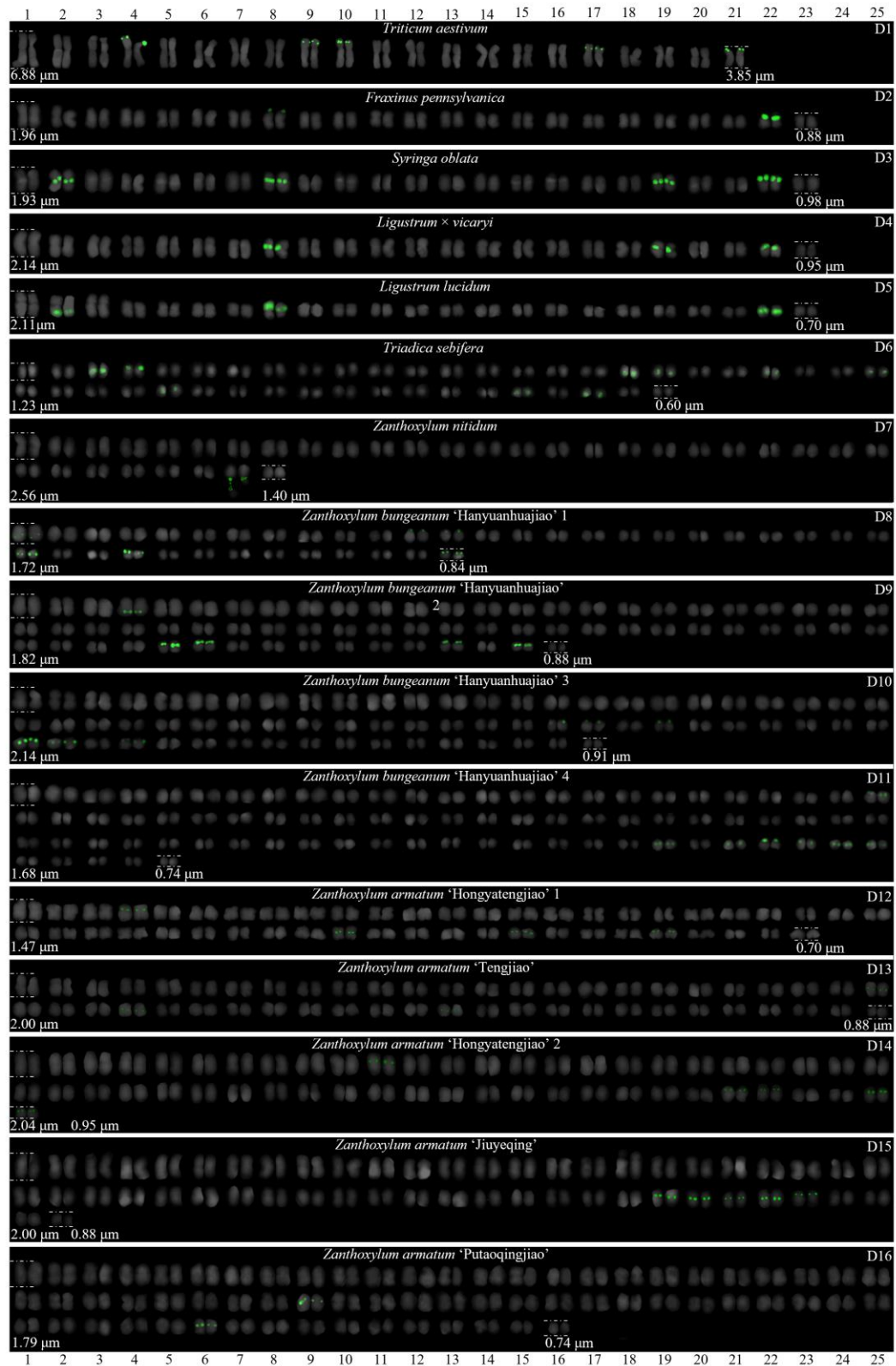

Supplement: Supplementary file 1 [file genes-15-00647-s001.zip › Supplementary Figure S4.pdf]

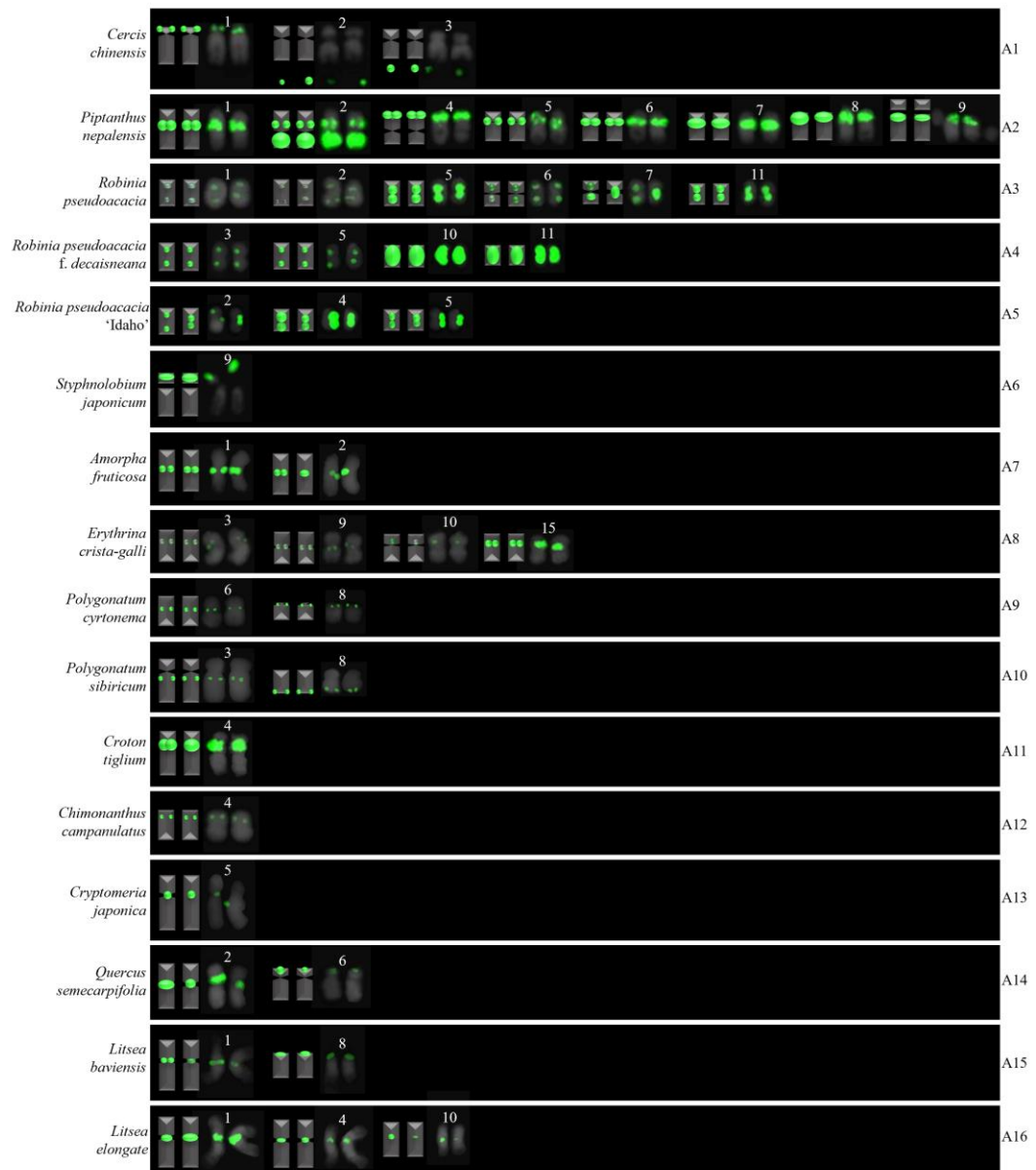

Supplement: Supplementary file 1 [file genes-15-00647-s001.zip › Supplementary Figure S5.pdf]

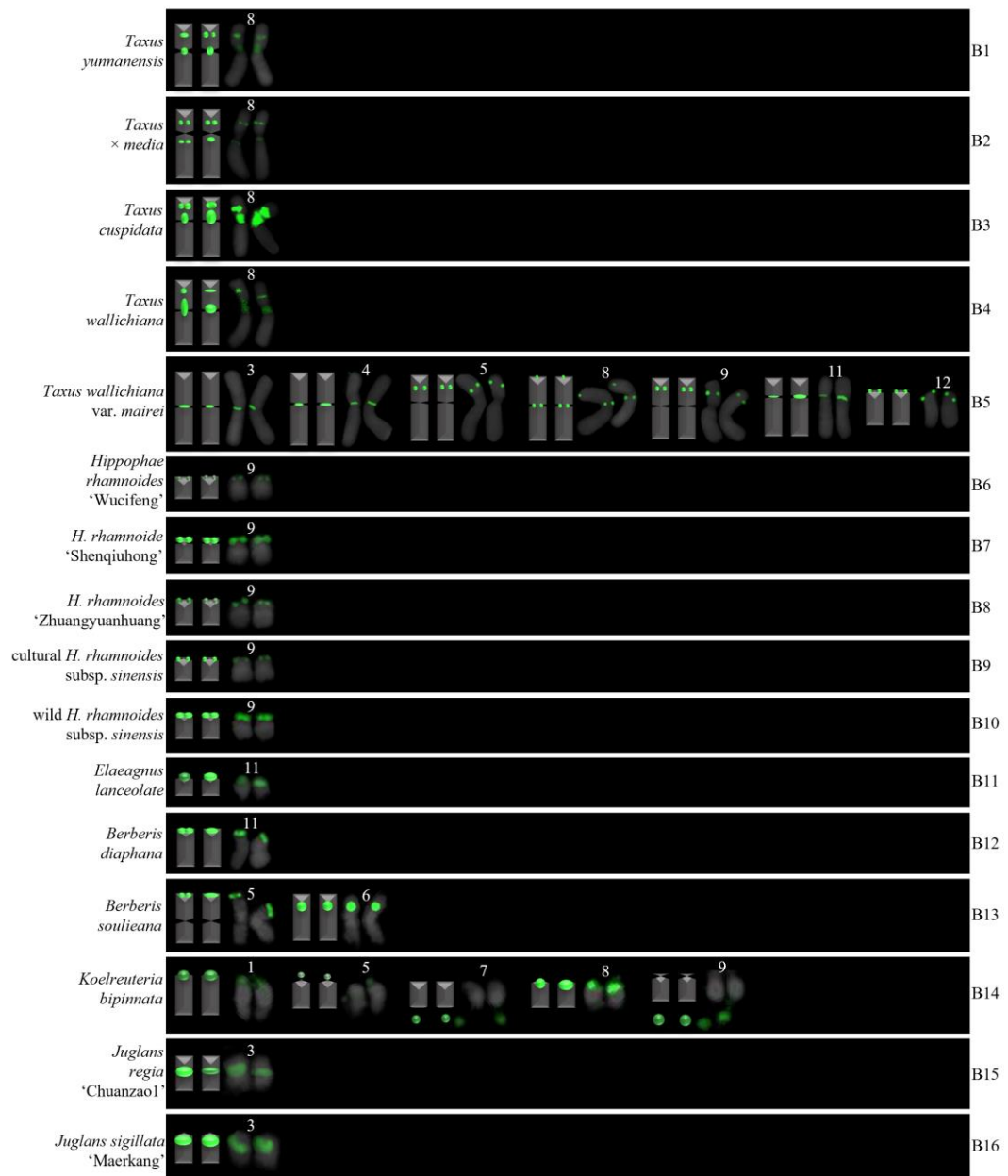

Supplement: Supplementary file 1 [file genes-15-00647-s001.zip › Supplementary Figure S6.pdf]

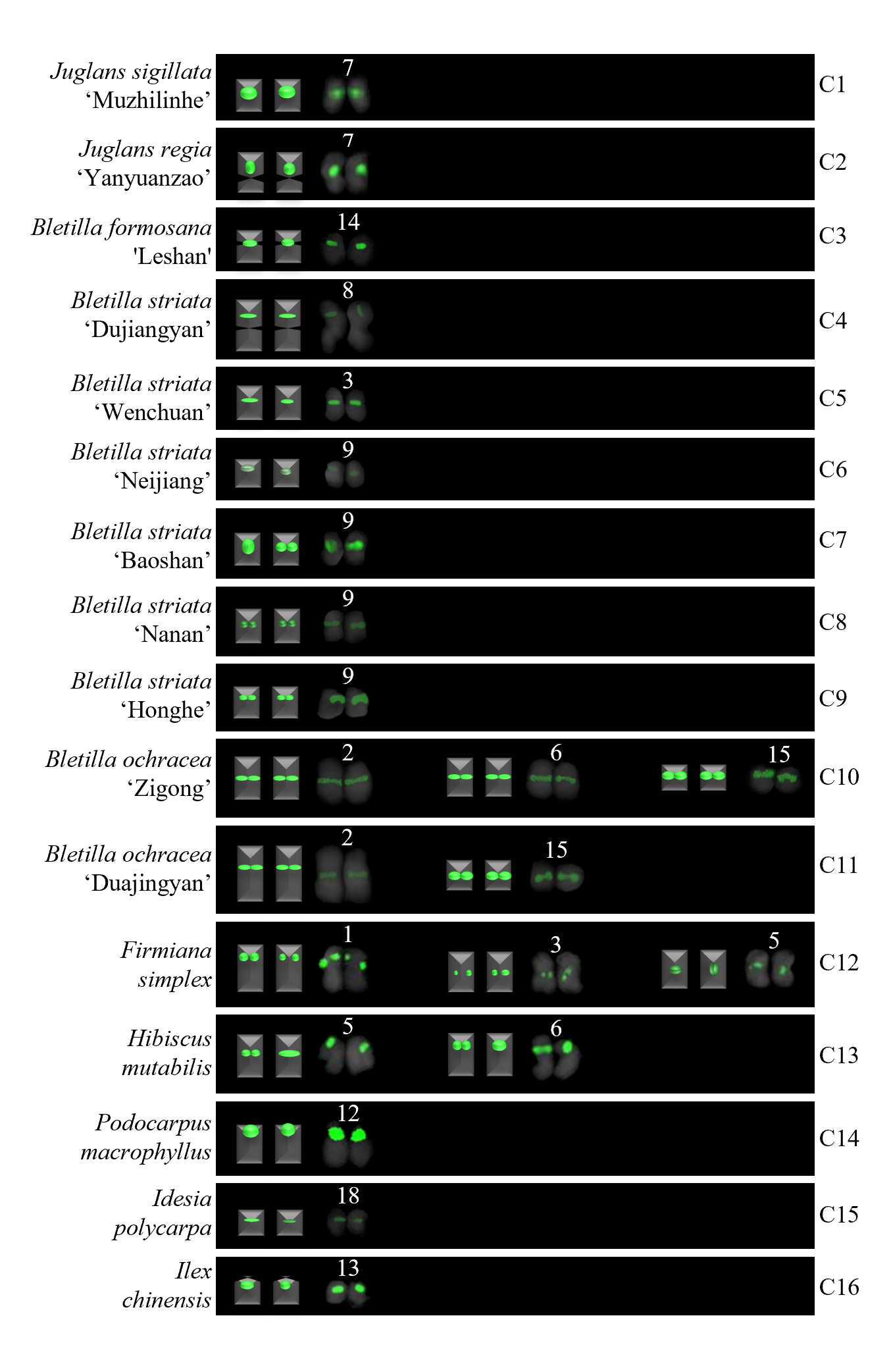

Supplement: Supplementary file 1 [file genes-15-00647-s001.zip › Supplementary Figure S7.tif]

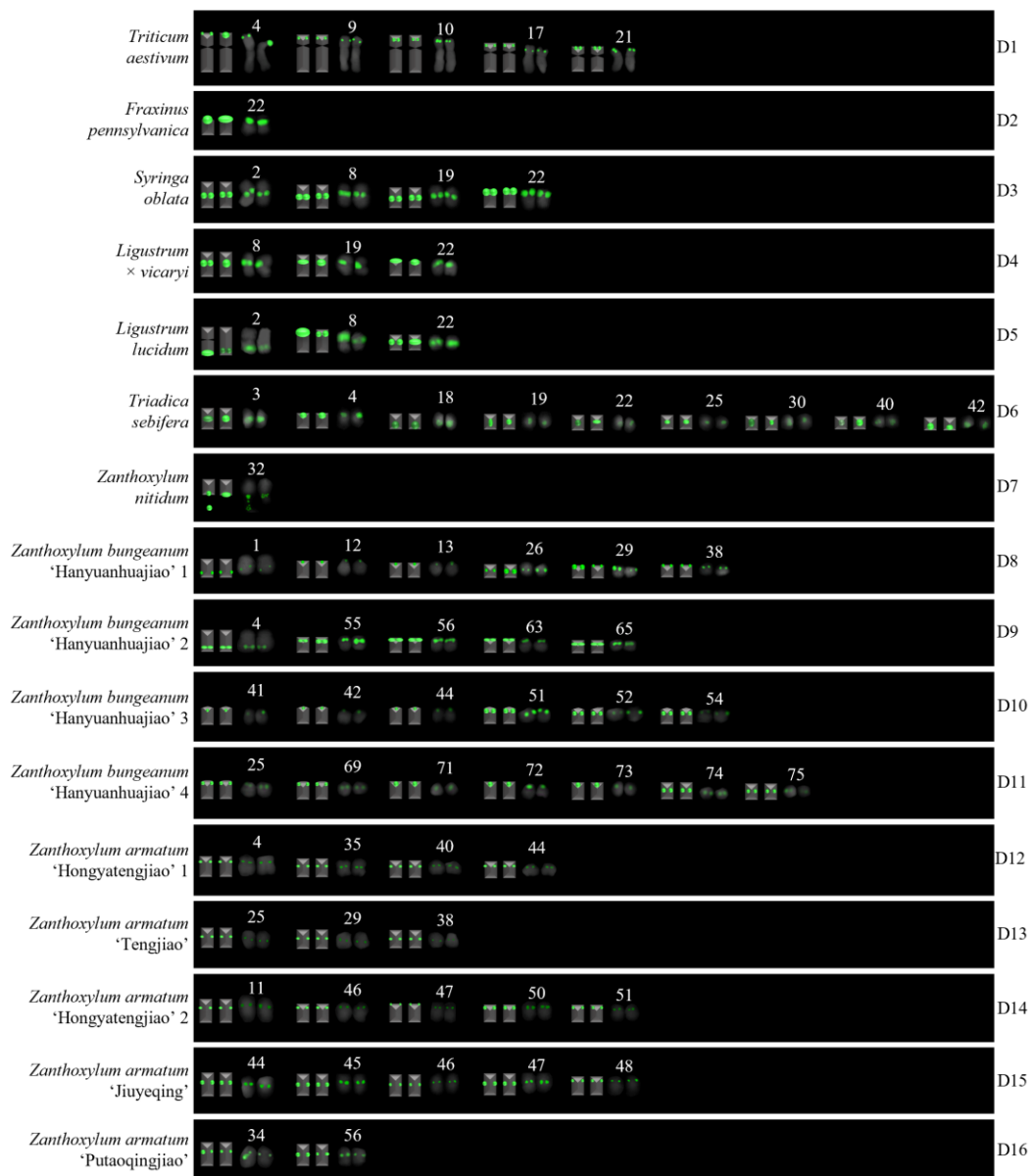

Supplement: Supplementary file 1 [file genes-15-00647-s001.zip › Supplementary Figure S8.pdf]
